# Using Non‐Standard Research Methods to Explore the Perspectives of People With Intellectual Disabilities on Sensitive Topics: A Discussion of the Research Paradigm, Data Collection Methods and Data Analysis

**DOI:** 10.1111/jar.70232

**Published:** 2026-04-29

**Authors:** Irene Tuffrey‐Wijne, Andrea Bruun, Rebecca Anderson‐Kittow

**Affiliations:** ^1^ Kingston University London London UK

**Keywords:** data analysis, death and dying, inclusive research, intellectual disability, interpretivist phenomenology, qualitative research

## Abstract

**Background:**

To make research on sensitive topics accessible to participants with intellectual disabilities, researchers have used innovative, non‐standard data collection methods. However, there is a lack of information about data analysis, validity and reliability.

**Methods:**

Using the example of a focus group study on end‐of‐life care planning with people with intellectual disabilities, the development and implementation of several creative data collection methods and the process of data analysis are described and discussed.

**Results:**

Newly developed data collection tools were presented as ‘games’ that successfully elicited participant perspectives. Data analysis involved the Framework method within an interpretivist phenomenological research paradigm. It incorporated extensive team discussions, including researchers and advisors with intellectual disabilities.

**Conclusion:**

Any activity can become a valid data collection tool if it fits the study's research paradigm, has a clear rationale, is co‐developed and tested with people with intellectual disabilities and if the analytical process is clearly described.

## Lay Summary

- We are a team of researchers with and without intellectual disabilities.
- People with intellectual disabilities are often not able to take part in research because the research methods (like interviews or questionnaires) are too complicated. If the research topic is sensitive (like death and dying), they are even less likely to be included in research.
- If researchers want to find out the views and experiences of people with intellectual disabilities, they may need to change the methods. In this article we describe how we used non‐standard, creative data collection methods. We use the example of a focus group study that explores the views of people with intellectual disabilities on end‐of‐life care planning. We called our methods ‘games’.
- It is important for researchers to show how they developed, tested and used their methods, and how they interpreted the results. They have to show that their findings and conclusions are not just the interpretations of a researcher, but as close as possible to the participants' own views.
- This article describes how we did this, working together as a team that included four researchers with intellectual disabilities.

## 1 Introduction

In this paper, we explore the use of non‐traditional data collection methods in sensitive research to enable inclusion of participants who have intellectual disabilities. Using the example of our own inclusive study on end‐of‐life care planning (Tuffrey‐Wijne et al. 2025), we focus on the rationale for developing and using non‐traditional data collection methods, as well as issues around data analysis, validity and reliability. We hope this paper will encourage further methodological debate and development.

### 1.1 Background

Following the slogan ‘Nothing About Us Without Us’, it is now accepted that policies and service delivery developments affecting the lives of people with disabilities must be informed by their perspectives (Harpur and Stein 2022). This has led to the development and growth of inclusive or participatory research paradigms, where people with intellectual disabilities are included as key contributors of knowledge (Walmsley and Johnson 2003; Nind and Vinha 2012; Chappell 2000). Qualitative health and social care researchers have grappled with significant challenges when including people with intellectual disabilities as research participants, especially if the topic is considered to be sensitive (McDonald et al. 2017; Tuffrey‐Wijne et al. 2008; Coons and Watson 2013). There are ongoing concerns around the vulnerability of participants with intellectual disabilities, ethics and consent (Northway et al. 2015; Lennox et al. 2005; Nicholson et al. 2013) which has led to a reliance on ‘gatekeepers’ and proxy reporting. A systematic review of studies focusing on palliative care of people with intellectual disabilities found that across all studies, involving 2970 participants, only 31 (1%) were people with intellectual disabilities themselves (Adam et al. 2020).

### 1.2 The Need to Use Non‐Traditional Data Collection Methods

Major barriers to inclusion are the cognitive and communicative abilities needed to cope with well‐established research methodologies, such as interviews and questionnaires (Cithambarm et al. 2021). Many people with intellectual disabilities have receptive and expressive language difficulties. This leads to obvious challenges if participants have difficulties in understanding or correctly interpreting the questions and coping with the research requirements (Lloyd et al. 2006; Hollomotz 2018). Early researchers using participatory research paradigms described some of these challenges, including the tendency of people with intellectual disabilities to acquiesce (answering ‘yes’ regardless of the question) and recency (selecting the last option) (Sigelman et al. 1981). They are often inclined to answer with a single word or short sentence rather than a free‐flowing conversation, needing a higher level of input and prompts from the researcher (Booth and Booth 1994). This could increase the risk of researcher bias.

To combat these methodological challenges, a range of adapted and creative data collection methods have been reported (Walmsley and Johnson 2003; Hollomotz 2018; McFarland et al. 2024; Bains and Turnbull 2022; Samways and Heslop 2025; Tuffrey‐Wijne et al. 2012). It might not be sufficient to make basic ‘reasonable adjustments’ to established research methodologies, for example by including the option of researcher prompts or pictures to support questionnaires or interview questions. A radically different approach to data collection might be needed. This fits a research paradigm based on the social model of disability, where non‐disabled structures must change to become accessible to people with disabilities and not the other way around (Matysiak 2001).

Within death and dying research, this has included the use of icebreakers, modifying the number and type of questions, using visual media to facilitate conversations, and role play (Diaz et al. 2024). Several studies have used pictures to tell a story related to the research topic. Pictorial storytelling was found to be effective in facilitating discussion and exploring opinions, emotions and experiences in groups of people with intellectual disabilities, with participants being able to identify with the story and project their own emotions and concerns onto it (Cithambarm et al. 2021; Tuffrey‐Wijne et al. 2012, 2007; Rodriguez Herrero et al. 2014; Reilly et al. 2020; McLaughlin et al. 2015; Voss et al. 2020). The Nominal Group Technique has been adapted by using images to illustrate ideas that participants could vote on, with cards and voting boxes (Tuffrey‐Wijne et al. 2012, 2007; Bekkema et al. 2016). A number of studies have used Talking Mats, a visual framework with picture communication symbols to aid communication and help people share their views on a given topic (Stans et al. 2019). Data collection methods that have enabled people with limited verbal ability to participate include Photovoice, which uses participant‐generated photographs and narratives to explore participant perspectives (Cluley 2017; Povee et al. 2014; Krisson et al. 2022; Rojas‐Pernia and Haya‐Salmón 2022; Chinn and Balota 2023; Chinn et al. 2024); and incorporating participants' drawings, music and poetry (Kennedy and Brewer 2016).

### 1.3 Lack of Reported Detail on Data Analysis

However, whilst a range of methodological techniques and approaches have been identified, many study reports lack methodological detail, in particular with regards to data analysis (Diaz et al. 2024; Hayden et al. 2024). This raises important questions about how researchers can demonstrate the validity, reliability and trustworthiness of their findings. We do health and social care research in order to generate understanding and insights, which will ultimately inform policy and practice and for other researchers to build on. This will not happen unless our study findings are seen as sound and believable by those whose services and policies we want to inform, including commissioners, service providers and professional bodies as well as research funders.

For researchers using inclusive research paradigms, there can be a tension around the desire to involve people with intellectual disabilities in the data analysis process, and the difficulties of doing so (Coons and Watson 2013). Some researchers have expressed concern that this may hold back the development of theory and practice, and that there should be a place for qualitative and inclusive researchers to theorise without the involvement of people with intellectual disabilities (Walmsley et al. 2004). However, there are examples of successful inclusion of the perspectives of people with intellectual disabilities in the data analysis process. Tuffrey‐Wijne and Butler (2010) describe a process of inclusive data analysis in a study where the researcher spent extended periods doing participant observation with 13 people with intellectual disabilities who were living with (and in most cases, dying of) cancer. Summaries of the field notes data were presented to a researcher with intellectual disabilities as ‘stories’ in vignette format. His comments and reactions to these stories were recorded, and his interpretations were compared across multiple team members without intellectual disabilities. This led to a re‐examination and adjustment of the final themes. It enhanced authentic analysis and increased the reliability of generated themes. Others have identified similar strengths of incorporating people with intellectual disabilities in the analytical process (Kramer et al. 2011).

#### 1.3.1 The Importance of Demonstrating Validity and Reliability

In qualitative research paradigms, validity means that we can feel confident about our interpretations and conclusions (Cresswell 2013). In other words, do our presentations of the findings match the participants' experiences? Are the findings plausible, trustworthy, and have a high level of credibility? To achieve this, chosen research methods need to be based on established rigour. Whilst clear methods have been established for analysing qualitative interview data that involve written words such as interview transcripts, how to ensure methodological rigour for creative and inclusive methodologies is less well described. A clear and detailed methodological description is therefore paramount.

Reliability concerns consistency and dependability in data collection, analysis and interpretation. This involves demonstrating that the methods used are appropriate, that the data are collected and analysed systematically, and that the interpretations are well‐supported by the evidence. Reliability is enhanced by establishing clear and consistent protocols for data collection, analysis and interpretation, a clear audit trail and transparent reporting (Miller 2008).

### 1.4 Aim

This paper aims to explore and discuss the rationale, development, implementation and analysis of inclusive, non‐standard, flexible data collection methods in sensitive research with people with intellectual disabilities. We use the example of a series of focus groups we conducted with people with intellectual disabilities to explore their views on end‐of‐life care planning, including when, how, and with whom end‐of‐life care planning should be done. These focus groups formed the first part of the Victoria & Stuart Project (https://www.victoriaandstuart.com/), a study we conducted between 2022 and 2024, aimed at co‐producing a toolkit of approaches and resources for end‐of‐life care planning within intellectual disability service (Tuffrey‐Wijne et al. 2025).

The objectives of this paper are to:

- Describe the research paradigm and epistemological stance that underpinned the focus groups;
- Describe the rationale, development, characteristics, implementation and outcomes of our data collection methods;
- Describe the process of data analysis and
- Explore the challenges around academic rigour, reliability and validity.

We use the COnsolidated criteria for REporting Qualitative research (COREQ) checklist (Tong et al. 2007).

### 1.5 The Research Team

The research team consisted of three postdoctoral researchers in the roles of project lead I.T.‐W., project co‐lead R.A.‐K. and project manager A.B. (this paper's authors). The team also included four researchers with intellectual disabilities who had no formal qualifications, a postgraduate researcher whose role it was to support them and a postgraduate project co‐manager. All, including the researchers with intellectual disabilities, were fully involved in developing and using data collection instruments and data analysis. Due to the abstract nature of this paper, it was written by the postdoctoral researchers only. However, we discussed writing a paper about our use of non‐standard data collection methods with the researchers with intellectual disabilities, and they were supported to write their own account of it (see File S1).

## 2 Study Methods

### 2.1 Design and Theoretical Underpinning

It is important to be clear about the study's underlying philosophical stance, as it affects the choice of data collection methods and the data analysis, as well as the role of the researchers (Savin‐Baden and Howell Major 2013). We rejected a positivist approach, which believes that there are objective truths that can be independently verified. Positivists rely heavily on objectivity and dismiss the importance of the subjective experiences and values of both the participants and the researchers (Park et al. 2020). Rather, like many researchers conducting qualitative research with people with intellectual disabilities, we subscribed to an interpretivist approach. This emphasises the importance of understanding the subjective meanings that impact the way people understand and interpret their world. The concept of subjectivity is central to this, as is the significance of understanding social and cultural contexts. Specifically, we adopted an interpretive phenomenological study design. This emphasises the study of people's lived experiences to understand the essence of a phenomenon (Smith and Nizza 2021)—in our study, the phenomenon was ‘planning ahead for dying’. There are no prescribed data collection methods within interpretive phenomenology. It requires a flexible approach, with methods adapted to capture the depth of people's experiences (Tavakol and Sandars 2025).

An acknowledgement of the existence of multiple perspectives is important and consistent with an interpretivist paradigm (Lincoln and Guba 1985). Interpretivist researchers cannot be independent of their own values and assumptions, which need to be consciously acknowledged and then ‘bracketed’ or set aside in order to focus fully on the participants' perspectives. However, in the analysis stage, researchers' own knowledge and values are re‐examined and utilised to help make sense of the participants' experiences and perspectives, and findings can be put into a wider social, cultural and theoretical context (Larkin et al. 2006). To enhance trustworthiness and reduce the risk of researcher biases, researcher reflexivity is crucial, with careful examination of personal biases, values and experiences (Olmos‐Vega et al. 2023). In reporting the study results, it is important to be transparent about the sources of new knowledge and describe clearly whether it comes directly from the participants, or from the researchers' interpretation (Smith et al. 2021).

### 2.2 The Focus Groups

A focus group methodology was chosen because in our previous experience of facilitating discussions with people with intellectual disabilities around death and dying, we found that being in a group can help people to communicate their views on sensitive or potentially upsetting topics (Tuffrey‐Wijne et al. 2012; Tuffrey‐Wijne 2013). Those less able or willing to volunteer experiences or opinions may contribute their thoughts and feelings through body language whilst they listen and observe, or by actively responding to others; people are often encouraged or inspired to speak by hearing others.

The groups were facilitated by one or two researchers with intellectual disabilities and between two and four non‐disabled researchers. We found that the facilitative power of the researchers with intellectual disabilities lay not in impartiality but in their ability to share of themselves within the group, to give their opinion about participants' contributions and to resonate with them. Unlike textbook guidance, which emphasises the importance of facilitator neutrality (Krueger and Casey 2015), in our focus groups, all facilitators consciously shared of themselves, commenting with recognition on participants' contributions. This validated participants' experiences and encouraged further contributions (Butler et al. 2012). It fits an interpretivist paradigm, where the researchers' and participants' perspectives and interpretations impact on each other.

#### 2.2.1 Sample and Setting

Participants had to be adults, have intellectual disabilities, have some verbal capacity and be able to give informed consent. They were recruited through our study collaborators, including intellectual disability service providers in England who acted as ‘gate keepers’, passing on study information in easy‐read and video format to potential participants. They also liaised with participants' support workers, who were additional gatekeepers with the potential to block participation if not well‐informed or supportive, but who could also be important facilitators helping participants feel confident to contribute. Informed consent was obtained by the researchers.

A total of 19 participants took part in four focus groups (between 4 and 6 participants per group). Participants within each group knew each other. Ages ranged from 20 to 60. Five participants were from minoritised ethnic communities and 14 were white. Each group also included several support workers who knew the participants and were able to provide personalised support. The groups met for two 2‐h sessions, either in 1 day or over two consecutive days. Three groups met in person in different parts of England and one group met online. All focus group sessions were video‐recorded and transcribed verbatim. In addition, one member of the research team wrote detailed field notes during the sessions.

### 2.3 Data Collection Methods: Rationale, Development and Implementation

#### 2.3.1 General Approaches and Principles

Over 15 years of conducting inclusive focus groups (Anderson‐Kittow et al. 2024; Tuffrey‐Wijne et al. 2007, 2012, 2013, 2020), we have found that participants can be nervous about not understanding, not knowing the answers or ‘getting it wrong’, and that there can be a desire to ‘fit in’ with the group consensus. There is also a power imbalance between participants and researchers, especially non‐disabled researchers (Coons and Watson 2013). We have developed several approaches and principles for combating some of these challenges. These include (Harpur and Stein 2022) having trained co‐facilitators with intellectual disabilities; (Walmsley and Johnson 2003) creating a non‐threatening and welcoming physical space with props, pictures, signs and refreshments; (Nind and Vinha 2012) re‐framing data collection activities as ‘games’ rather than interview questions; (Chappell 2000) facilitators demonstrating or taking part in group activities and sometimes deliberately struggling or presenting contrasting views; (McDonald et al. 2017) using music and movement; stressing that there are no wrong answers; and (Tuffrey‐Wijne et al. 2008) giving participants a round of applause for being brave and honest if say they do not know something or if they express a view that is different from everyone else's. The overall aim is to create a space of safety where participants feel important, listened to, supported and able to share their true thoughts and feelings.

#### 2.3.2 Data Collection Methods

Most data collection methods, including ice‐breakers, were introduced as ‘games’, including the Name Game, the Hat Game, the Box Game and the Washing Line Game (see descriptions below). The first three games were piloted before the start of the study with 16 people with intellectual disabilities in three in‐person groups and refined with two researchers with intellectual disabilities. The Washing Line Game was conceived and trialled with the researchers with intellectual disabilities. The initial plan to include standard conversational techniques in the focus groups (asking open questions without additional activities or visual aids) was discarded, as this did not elicit useful answers in the pilot groups. This confirmed conclusions from an earlier focus group study that even if participants have a reasonable level of verbal skill, simply asking questions is not sufficient, and additional research techniques are needed to elicit the necessary data (Tuffrey‐Wijne et al. 2012).

#### 2.3.3 Starting and Ending the Session

We started each session with around 20 min for ice‐breaker games. Everyone took part, including researchers and support staff. The purpose was to settle any nerves and break down barriers between participants and researchers. Crucially, the first part of these ice‐breaker games had nothing to do with the difficult research topic. It was important to create a safe space first. Sessions started with the ‘Name Game’, which involved throwing a soft toy to another person whilst calling out (or asking for) their name. This often resulted in cheers for good catches or hilarity about missed ones, providing the first opportunity to break down power structures. For the Hat Game, we sat around a table displaying a diverse selection of hats and wigs; for the online meeting, we asked people to bring a hat to the screen. Nerves tended to make place for laughter as people saw everyone, including the researchers, looking rather silly. We asked ‘Put on your hat if…’ followed by a range of questions (e.g., ‘you like Abba’, ‘you came here by bus’). This allowed us to go round and ask people to expand, with the purpose of getting everyone used to speaking in the group with a non‐threatening question (‘Which Abba song do you like?’ or ‘You don't have a hat on, tell me what music you DO like?’). We found music questions particularly useful, as favourite songs can be quickly found on YouTube and played out loud; this helped to support nervous or less talkative participants. Once nerves were settled and participants seemed relaxed, the final hat question was asked, which led into the research topic: ‘Put on your hat if you have ever been to a funeral’. There was never a hatless person following this question. It let participants know that difficult experiences around death and loss were shared by everyone in the group, including support workers and researchers.

Taking 15 min to end the session was equally important. In two round‐robins, people held a soft toy whilst they spoke. Starting with ‘How am I feeling now?’ a researcher role‐modelled possible answers, for example ‘I am feeling happy to have met you all and listen to your stories, but also a bit sad because John's story made me sad’. The second round‐robin was light‐hearted and positive, on a topic not related to dying; for example, each person saying what they are looking forward to in the week ahead.

#### 2.3.4 Pictorial Story Telling

We showed selected images from *Am I Going To Die?*, a pictorial story by Beyond Words (Hollins et al. 2009) about ‘Bob’, a terminally ill man. The dual purpose was to introduce the concept of dying and the different decisions to be made in the final months of life, in preparation for subsequent group activities; and to begin eliciting participants' views on different aspects of end‐of‐life care planning.

#### 2.3.5 The Box Game

The research question behind the Box Game was: ‘Would you be happy to talk with someone about…’ with regards to a range of end‐of‐life care planning choices (see Box 1). The Box Game involved three colourful boxes with large emojis, representing yes/no/not sure choices: smiley/thumbs up (green), sad/thumbs down (red) and frowning/thinking (blue). Participants were given a set of pictures with captions and invited to deposit each in the box of their choice. In the online group, participants had been sent paper versions of the yes/no/not sure pictures, which they could hold up to the screen in response to the questions.

Before starting this activity, we gave participants some non‐threatening practice questions, to get used to the process and take away their worries that research activities are too difficult. Practice questions included: ‘Do you like… Pizza? Spiders?’ and ‘Would you like to decide for yourself… What food you eat? Where you go on holiday?’ Participants enjoyed this; they appeared empowered and got a confidence boost from being able to express their opinions with actions rather than words.

Each participant was supported individually, either by a researcher or their support worker, to decide which box to put their pictures in. Any further details or thought processes given by the participant were written on the card by the supporter. As much as possible and practical, these discussions were also captured in field notes by researchers.

The Box Game provoked sometimes strong and clear responses. One participant put the ‘Planning my funeral’ image firmly in the NO box:

*Participant X:* Oh God! I don't want to talk about that yet; the funeral. It's not for me yet; the coffin.

The same participant had put the ‘What will happen when I die’ image in the YES box:

*Participant X:* Choose with help; carer will help.

However, in the same group, another participant was clear that she did not want to engage with any of the images, not even to put it into the NO box. This prompted the research team to ‘think on their feet’ and provide the participant with a physical bin for the picture. (This ‘bin’ became a permanent feature of the box game for future focus groups.) Participants were supportive of each other's various choices:

*Participant Y:* Don't want to talk about this question. *[Rips up the image and puts it in the bin]*.

*Participant X (addressing Participant Y):* You did the right thing. It's not for you now. It's not for me yet. I'm not going to die.

The activity also prompted group discussions, where participants shared their thoughts with the whole group. For example, the question ‘Who speaks for me’ had an almost unanimous response that this should be someone you trust, naming family members or specific staff members; they were clear about which staff they did or did not want to involve. Others reflected on not having a trusted person in their life, or the fact that the end of life is some time in the future, when support workers are likely to have moved on. The following discussion was captured in the online focus group recording:

*Participant A:* I don't have that. I used to have some, so some (…) I have lost a care worker who I'm very, very well with and who's just moved.

*Researcher:* Do you think they could be part of helping you, still part of helping you decide these big things about your life, or is that not possible?

*Participant A:* I wouldn't say it's possible because they got their own lives to lead, so it's so sorry (…) Because they moved on. They got different jobs. They got different responsibilities. So we can't expect it.

(…)

*Participant B:* If that person's no longer paid to be there, why should they be there? (…) That hurts a little bit. But I can understand. It's a job.

#### 2.3.6 The Washing Line Game

The Washing Line Game was developed to help participants consider the question ‘When should you start end‐of‐life care planning?’ It was an attempt to make the abstract concept of time more tangible and visual. A long line was strung across the length of the room, and 11 A4‐sized images were pegged onto it, depicting the life of ‘Bob’ from the earlier *Am I Going To Die?* story. It included images of Bob as a child, moving into his own home, seeing a doctor, going into hospital, being very ill in bed, dying and having his funeral. We gave participants the same images we had used in the Box Game and asked them to peg each one onto the washing line at the place where they thought people should start to think and talk about it. Each participant was supported individually to think through each image. Any verbatim quotes were written on the back of the card by a supporter. For the online group, an image of the washing line was created and shown using Miro software, an online interactive whiteboard that allowed the facilitator to zoom in on different elements. The imagery helped people understand the question and prompted relevant discussions.

We found that most participants positioned a range of end‐of‐life care planning conversations on the timeline before Bob was very ill and dying, either before seeing the doctor or around the time he was in hospital:

‘Before I am too poorly’.

However, most did not think they themselves had reached that point yet:

*Participant X: ‘*In the middle. Not just now’.

*Participant Z: ‘*I've got the rest of my life ahead of me (…) I would start thinking about that not yet because I'm still young and it's very early’.

One young adult, who had already been asked about his end‐of‐life care plan by his support staff, explained:

*Participant B:* They said to me, have you thought about how you're gonna die, and I just said, Well, I haven't thought how I'm going to live yet! Let me get a little bit older, you know. (…) What happens when I get to 79 and all the things I've put at 29 have changed?

### 2.4 Data Analysis

#### 2.4.1 Framework Analysis

Data analysis is a complex, continuous iterative process that starts with forming ideas at the beginning of a study. The focus group data were analysed in conjunction with data from the wider study, which involved focus groups with a range of other stakeholders (i.e., family carers, support workers, service managers, healthcare professionals and policy makers). Throughout the data collection period, we used the framework analysis method (Gale et al. 2013). This seeks to identify commonalities and differences clustered around themes, which can be used to draw out descriptions or explanations. It is an often‐used method by qualitative researchers, which we simplified to make it easier for non‐expert researchers to contribute to. We created a matrix of rows of ‘cases’ (one for each focus group) and columns of ‘codes’ representing the key themes or areas of investigation. This produced cells for summarised data. This was mostly deductive coding, with pre‐determined themes which had been derived from the literature and from the extensive stakeholder consultations that led to the study's protocol development. Starting with deductive coding was helpful as it gave a clear framework to start discussions with intellectual disabilities. However, there was also space for inductive coding, where new themes could be found within the data and added to the framework, leading to new insights. We found this method to be understandable to researchers with intellectual disabilities as well as to the many non‐academic stakeholders who were involved in making sense of the data, including families and support workers. The analytical method also fitted well with our phenomenological research paradigm.

Framework analysis is typically used with text‐based data, in particular interview data. It starts with verbatim transcription and familiarisation before line‐by‐line reading and systematically applying codes to the text. These are then ‘charted’ into the framework matrix, which involves summarising the data by category (Gale et al. 2013). Whilst this worked well with the other focus groups in the wider study, relying on focus group transcripts and field notes alone did not do justice to the richness of data from participants with intellectual disabilities. Although the transcripts and field notes were invaluable aide‐memoires, many of our insights came from interactions that were less easy to ‘catch’ by microphone or pen. Our analytical method, therefore, mostly involved team discussions and reflections. Immediately after each focus group, and again during focused data analysis sessions, the researchers (crucially, including those with intellectual disabilities) held extensive discussions about the participants' responses with regards to the framework's codes, but also allowing for new topics or insights. A summary was then ‘charted’ and added to the relevant framework cell. See Box 2 for an example of extracts from the charted data.

#### 2.4.2 Example of Data Analysis Through Team Discussion and Reflection

The team reflected extensively on the contributions of one participant who repeatedly indicated she did not want to think or talk about dying. She sometimes removed herself from the group by sitting close to the door but did not take up the researchers' suggestion that she could leave, and returned for the second session. Despite putting all her images in the bin during the activities and stating explicitly that she did not want to talk about dying, she contributed many thoughts about Bob's story. Having refused to talk about ‘What I want to do before I die’, she was keen to respond when we re‐phrased the question as ‘What I want to do when I am still alive’. This led to team reflect on the barriers of death‐related language and the importance of finding ways to enable people to communicate how they want to live (even if they only have a short time left to live), rather than how they want to die.

#### 2.4.3 Incorporating a Range of Views and Perspectives in the Analysis

To arrive at an interpretation that accurately reflected participants' experiences, we presented and discussed the framework summary with an advisory group of 24 stakeholders, including three people with intellectual disabilities. The next step was to work with two co‐design groups of stakeholders to develop a toolkit of end‐of‐life care planning resources, building on the focus group findings. We summarised the ‘framework’ for them in the form of a video compilation of actual focus group footage (with participants' permission) clustered around the framework's themes. The creation of the video compilation as a starting point for the co‐design process was part of the study's Experience Based Co‐Design methodology (The Point of Care Foundation 2018). It helped to ground our reflections and work in the participants' experiences and views. One of those co‐design groups consisted of eight people with intellectual disabilities, five of whom were purposively selected from the focus groups, inviting those who had been highly engaged with the activities although not necessarily keen to do end‐of‐life care planning. This lengthy process of reflecting on the focus group data, involving dozens of team members and collaborators, ensured that our interpretations of the study findings are both valid (providing a deeper understanding of the issues, moving beyond participants' contributions whilst faithfully presenting the participants' views) and reliable (analysed systematically, in a clear and transparent manner).

#### 2.4.4 Integration of Findings

The final steps in the data analysis process were to integrate the findings and themes from all the focus groups (including those with other stakeholders), incorporating the reflections and feedback of research team members, advisors and co‐production group members. The final themes were defined, named, written up and published (Bruun et al. 2024). These final steps required significant skills in abstract thinking and theorising and were completed by A.B. with the support of the other post‐doctoral researchers on the team.

File S2 gives a 10‐step overview of how the analytical process was operationalised for this study.

## 3 Discussion

The experiences and perspectives of people with intellectual disabilities are unlikely to be captured in the participants' words alone, but may be presented through actions, silences, smiles, body language and behaviours that need careful interpretation. We argue that it is important, and indeed essential, to deviate from conventional, rigidly prescribed data collection procedures to elicit their experiences and perspectives. Any activity could become a data collection activity, as long as the researchers have a clear rationale for it, have co‐developed and tested it with people with an intellectual disability, and have a transparent plan for collecting and analysing the data. Data will need to include field notes capturing both the researchers' observations and their immediate reflections.

We believe that including people with intellectual disabilities as part of the research team is essential for studies that try to capture and understand the experiences and perspectives of people with intellectual disabilities. This is a skilled role that requires time, training and resources. Bringing in a researcher with intellectual disabilities only for specific data collection activities, such as co‐facilitating a focus group, is not sufficient. They need to be part of the team from the beginning to co‐develop and test the activities and understand what it is like to do them. They also need to be part of the data analysis process because their perspectives on the data will increase the trustworthiness and validity of the findings (Tuffrey‐Wijne and Butler 2010).

### 3.1 Reflecting on the Use of Unconventional Data Collection Activities

Reframing our data collection activities as ‘games’, allowing plenty of time for ice‐breakers, and consciously validating any contribution made by participants (including silences, objections and withdrawals) helped participants to feel safe. All participants indicated that they had enjoyed the sessions, despite the challenging topic. We had the impression that many of the participants were not used to feeling ‘heard’ or having their opinions sought, listened to and validated. This was a positive and empowering experience for them.

Our data collection activities were effective in exposing participants' feelings and emotions around end‐of‐life care planning. For example, one participant's simple words (‘I don't want to talk about dying’) obscured a more complex picture. Her actions of ripping up death‐related images but remaining part of the group and contributing to discussions revealed her keenness to engage and be ‘known’ with regards to important life decisions, but not necessarily through talking about them.

Our ‘Box Game’ activity may be seen as a non‐paper version of a tick‐box questionnaire or Likert scale. We used this qualitatively, as we were interested not only in participants' actual choice, but also in their thoughts and discussions during this process. It helped us to explore their perspectives in‐depth, as well as understand some of their contexts, in line with our interpretivist research paradigm. Similarly, the ‘Washing Line Game’ helped participants to understand and visualise and abstract questions about time. However, the key data we used in the analysis were not the positions they chose on the washing line for particular aspects of end‐of‐life care planning, but their comments and reflections on why they chose these particular points in time.

Other researchers have similarly experimented with developing new approaches to data collection (Rojas‐Pernia and Haya‐Salmón 2022; Coffey 2011). Nind and Vinha used stimulus materials, metaphors and poetry to aid expression, understanding of ideas and stimulate conversations. We support their argument that ‘moving away from pure talk might help to facilitate their [people with intellectual disabilities] active engagement in discussion’. (Nind and Vinha 2016) (p. 14).

### 3.2 Analysing Unconventional Data Sets

Starting the process of analysing unconventional data sets with team discussions and reflections was effective and allowed for the inclusion of hard‐to‐code data. It also allowed for the inclusion of researchers with intellectual disabilities in data analysis, who were unable to write field notes themselves. These discussions served not only to reflect deeply together on what we thought the participants tried to convey, but also to illuminate our own knowledge, values and ideas, bringing these back into the analysis. This analytical approach reflects the core tenet of interpretivism, emphasising the importance of understanding participants' perspectives in the context and circumstances of their lives, which they communicated to us through the research activities. However, deeper insights can be obtained by comparing their experiences and responses with our own and within the broader context of other data gathered in the wider study. It is important to acknowledge that our interpretations were grounded in the participants' contributions, but the wider interpretations and conceptualisations were our own.

### 3.3 The Challenge of Including People With Severe and Profound Intellectual Disabilities

The methods we described in our example would be inaccessible to people with severe and profound intellectual disabilities, who are at greatest risk of being excluded as research participants. For people whose cognitive and communication abilities exclude the use of images or intentional actions in response to requests, there are very few data collection methods described by researchers. Promising developments include the adaptation of using Photovoice, where participants with severe and profound intellectual disabilities are supported to show their perspectives through photography (Cluley 2017). However, the main way researchers have tried to access their perspective on the world is through ethnography, using participant observation (Redmore 2024), conversation analysis (Nicholson et al. 2021) or proxy reporting (Watson et al. 2017). These traditional methods have gathered useful data, but in order to give people the chance to express their own views, rather than relying on researcher observations, more creative methods need to be developed.

## 4 Conclusion

We have described the rationale, development and implementation of several creative data collection methods that served as data collection tools. These data collection tools were developed in order to encourage and enable people with intellectual disabilities to share their thoughts about the sensitive topic of death and dying. Data analysis involved a simplified form of framework analysis that was understandable to less experienced researchers and stakeholders. This approach was particularly suited to an interpretative phenomenological study design.

We believe that any approach or activity could become a valid data collection tool. We encourage other researchers not to shy away from developing their own creative data collection techniques, adapted to fit their participants and research questions. Important aspects of ensuring the study remains methodologically sound include: (1) co‐developing and testing/piloting the data collection method with people with intellectual disabilities; (2) making explicit what the actual data consists of—this is likely to include not only recordings but also field notes and researcher reflections; (3) involving a range of researchers and stakeholders in the process of reflection on the data, including people with intellectual disabilities (fitting the interpretivist paradigm) and (4) creating a clear analytical trail, including records of discussions and reflections within research teams and with stakeholders.

We encourage researchers to publish their approach, discussing the challenges and any perceived failures as well as successes. We welcome further debate on the methodological issues raised in this paper.

BOX 1 **Questions for the box game.**

Would you be happy to talk with someone about…

- What will happen when I am ill and dying
- Things I want to do before I die
- Who visits me
- Who will speak for me
- Where I want to be looked after
- If I'm going to have an operation or not
- Who is going to look after me?
- Planning my funeral

BOX 2 **Framework analysis: extracts from charted data.**

*Row: Focus Group 3*

*Column: ‘*Who should be involved’

[SUMMARY WRITTEN BY PROJECT LEAD, FOLLOWING TEAM DISCUSSION]: They wanted to make their own decisions, but also to be able to talk it through with a trusted person. For most this was a support worker they were close to; could also be family. They realised that if/when they can't speak for themselves anymore, others would have to speak for them. They wanted this to be several people—ideally a ‘circle of support’. They mentioned family and specific trusted support workers; it could also include a doctor or neighbours, for example. They all said that they currently had a circle of support, but *(our interpretation)* we think they meant that they KNEW who they would want to be their support‐decision makers, without this currently being written down or formalised. One participant said that ‘if you are a good friend to me, you are in my circle’—that is, all their friends are in their circle of support. Shared experience that you can get very close to a support worker: if they have supported you for years, they know you better than anyone. Participants wanted those support workers to stay in touch after they leave, and would attend ‘my funeral’, but weren't sure this could happen: support workers have their boundaries, they move on; if the person is not paid, they don't care anymore—that hurts.

*Row: Focus Group 3*

*Column: Team Reflections*

[PROJECT LEAD]: Struck by the importance of circle of support. How everyone knows who they wanted, but this did not seem formalised, so I'm not sure whether and how that can be tapped into/utilised. Key question is how you make important life choices in general—this is then extended into end‐of‐life choices, but fundamentally the same process.

[SUPPORT RESEARCH ASSISTANT]: Importance of LOVE. People wanted and needed this. Are support workers allowed to be true friends, or limited/have their wings clipped by boundaries?

*Row: Focus Group 1*

*Column: Team Reflections*

[RESEARCHER WITH AN INTELLECTUAL DISABILITY]: Participants were varied and individual. We can combine light‐heartedness and seriousness. Things can be untraditional. We learn something new from everyone.

[PROJECT LEAD]: WHO seems the most important question. Participants have such clear ideas about who should help them make decisions; including who could make the decisions that they didn't want to make themselves.

[PROJECT MANAGER]: Importance of respecting people's limits. We may not realise or recognise what the barriers are (e.g., female vs. male doctor might be easily missed as the reason for not wanting to talk to someone). What are the other obstacles?

## Funding

The study was part of a research project funded by the National Institute for Health Research (NIHR) Research for Social Care (RfSC), Research for Patient Benefit (RfPB) Programme (NIHR202963).

## Ethics Statement

The study received ethical approval from the West Midlands—Coventry and Warwickshire Research Ethics Committee (22/WM/0026) on 22/04/2022.

## Consent

All study participants were supported to understand an easy‐read Participant Information Sheet and provided informed written consent to participate in the study.

## Conflicts of Interest

The authors declare no conflicts of interest.

## Supporting information


**Data S1:** jar70232‐sup‐0001‐SupinfoS1.docx.

**Data S2:** jar70232‐sup‐0002‐SupinfoS2.docx.

## Data Availability

The final toolkit and its resources are freely available online on the study website: www.victoriaandstuart.com. Descriptive metadata are available on the Kingston University London Research Data Repository in 2024. Full data are not available, as they are not anonymised. Other relevant and shareable study data are presented in the study's published outputs: https://www.victoriaandstuart.com/publications.
