# Supplementary material for: Using Non‐Standard Research Methods to Explore the Perspectives of People With Intellectual Disabilities on Sensitive Topics: A Discussion of the Research Paradigm, Data Collection Methods and Data Analysis
Source: J Appl Res Intellect Disabil. 2026 Apr 29;39:e70232. doi: 10.1111/jar.70232 (PMC13128306; doi:10.1111/jar.70232)
Supplement: Supplementary file 1 — Data S1: jar70232‐sup‐0001‐SupinfoS1.docx. [file JAR-39-e70232-s002.docx]

**Supplementary File 1**

**Presentation of the focus group study and reflections on this paper by four researchers with intellectual disabilities***[4x AUTHOR NAMES WITHHELD]*

**What is this about? Why is this important?**

We are four researchers with an intellectual disability, working in a university team. Our research is about planning for when you die. This is important, because people with intellectual disabilities don’t always get the right care when they are dying. Often, they are not told about dying. They are not involved in making choices. We wanted to know if people with intellectual disabilities want to be involved in planning for dying. We wanted to know who else should be involved, when people want to start planning, and how to do it. Death is a very difficult topic to talk about. This article is about how we found out the answers and how we made sense of what we found.

**What we did**

We had focus groups with 19 people with intellectual disabilities. We then used some of our methods in a co-production group with 9 people with intellectual disabilities. We tried to talk about dying in a light-hearted and fun way. We developed three ways which we called “games”:

- story-telling with pictures
- a washing line with a life-story
- voting boxes with pictures

**What we found out**

The games helped everyone to talk about their feelings and opinions. Some people wanted to be involved in planning for their own end of life, and some didn’t. The games made people think about whose support they wanted to do end-of-life planning, when and how it’s best to do it. They liked activities where they didn’t need to write anything down.

**What does this mean?**

Research methods usually involve words, like interviews or questionnaires. We found that using games and pictures is also a good way of getting answers. It is important to try new ways like this, especially around scary topics, because it helps people with intellectual disabilities to be involved and give their opinions. It makes us equal.

It is important to involve us (researchers with intellectual disabilities) because the way we see things is not the same as everyone else. We see things at a deeper depth. We take things personally. We help you see things the way we do.
