# Supplementary material for: Using Non‐Standard Research Methods to Explore the Perspectives of People With Intellectual Disabilities on Sensitive Topics: A Discussion of the Research Paradigm, Data Collection Methods and Data Analysis
Source: J Appl Res Intellect Disabil. 2026 Apr 29;39:e70232. doi: 10.1111/jar.70232 (PMC13128306; doi:10.1111/jar.70232)
Supplement: Supplementary file 2 — Data S2: jar70232‐sup‐0002‐SupinfoS2.docx. [file JAR-39-e70232-s001.docx]

**Supplementary File 2**

**Analytical process for the [PROJECT NAME WITHHELD] focus groups**

1. Focus groups were held with people with intellectual disabilities families, support staff, service managers, palliative care professionals, and policy makers.
2. Ongoing framework analysis (alongside the focus groups) started after the first of the focus groups, conducted with the research team (including 4 researchers with intellectual disabilities). This included an account of the data provided by the researchers who had been present at the focus group, in order to (a) give all team members an overview, (b) begin discussions and reflections on the data, and (c) inform what content to include in the trigger film for the co-design groups.
3. Initial coding framework developed by the project lead [AUTHOR 1] to be used to analyse focus group data (document provided to the team, including detail of what each code covers) (deductive coding). These initial codes are based on relevant literature and experience with conducting end-of-life research with people with intellectual disabilities.
4. Further analytical discussions with the focus group facilitators [mainly AUTHOR 1 and AUTHOR 2] and the research team (including research team (including researchers with intellectual disabilities) were held throughout the process.
5. Throughout the process of facilitating and discussing the focus groups, four overarching themes/areas were identified. The results indicated that participants talked about these four interlinking areas related to end-of-life care planning: Funeral planning, life planning, illness planning, and talking about/understanding death and dying.
6. The four areas were discussed with the research team, the wider team of research collaborators (including the intellectual disability service providers who acted as gatekeepers) and the Research Advisory Group (consisting of a wide range of stakeholders, including people with intellectual disabilities, families, carers, professionals and policy makers). The themes resonated with people and it was agreed to continue working with this new framework.
7. The updated framework (the four areas of end-of-life care planning) was used in co-design trigger film and subsequently validated by co-design group members; it made sense to them.
8. Based on the knowledge gained in the focus groups and after validation (research team, wider collaborating team, Research Advisory Group and co-design group), the initial coding framework was updated to include the end-of-life care planning framework with the four areas (inductive coding).
9. The framework was updated by the project lead [AUTHOR 1] and project manager [AUTHOR 2].
10. The findings were written up [led by AUTHOR 2] and published.
